# Supplementary figures and images for: Leveraging a gain-of-function allele of Caenorhabditis elegans paqr-1 to elucidate membrane homeostasis by PAQR proteins
Source: PLoS Genet. 2020 Aug 4;16(8):e1008975. doi: 10.1371/journal.pgen.1008975 (PMC7428288; doi:10.1371/journal.pgen.1008975)

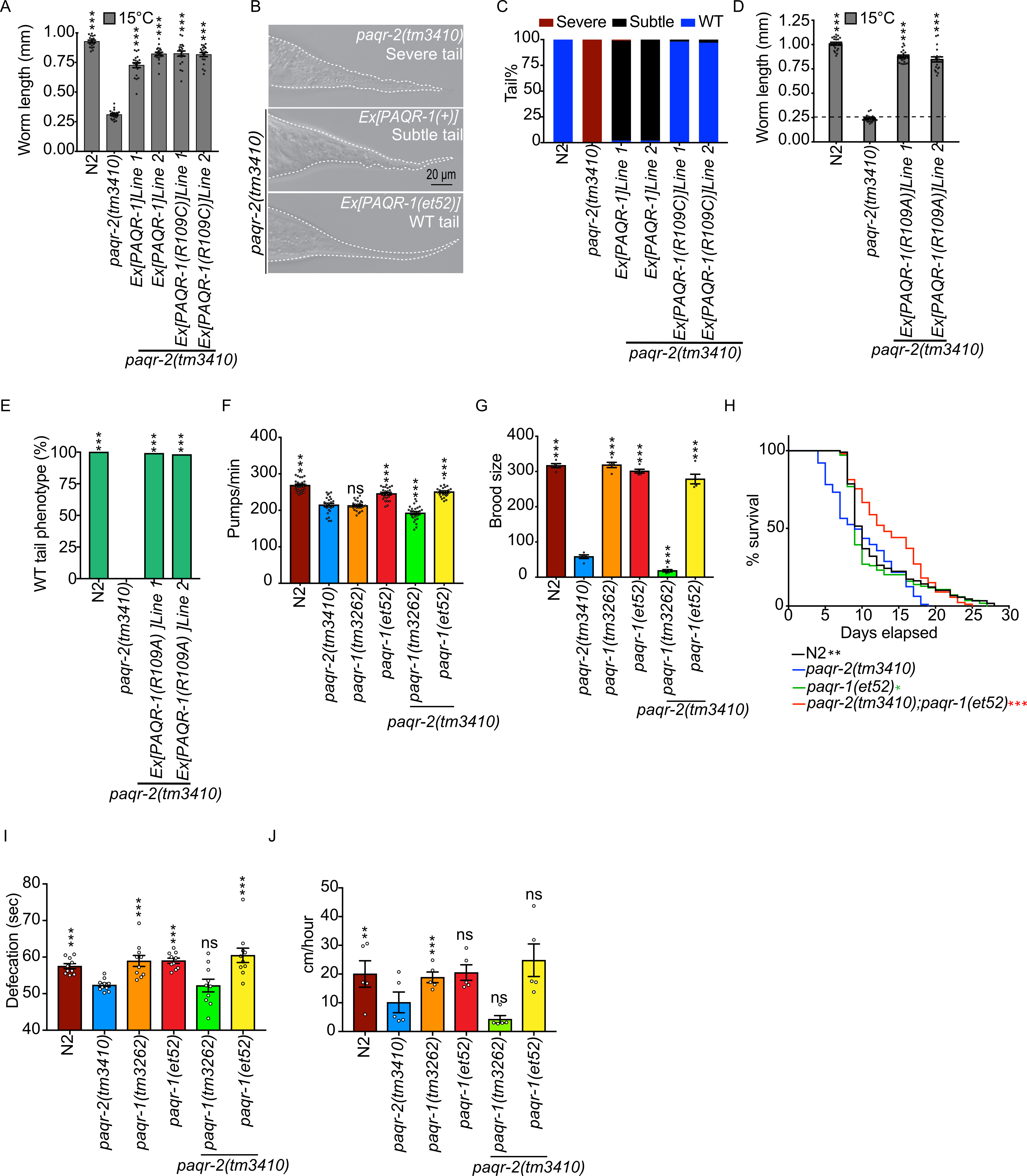

Supplement: S1 Fig — Several phenotypes were scored for worms of the indicated genotypes. (A) Length of worms placed as L1s on NGM media and measured after 144 hours cultivation at 15°C; (B-C) tail tip phenotype scored on 1-day old adults; (D) Length of worms of the indicated genotypes and placed as L1s on NGM media and measured after 144 hours cultivation at 15°C. Note that both paqr-2 mutant transgenic lines carrying the PAQR-1(R109A) expression construct are glucose and cold tolerant, and have normal tails. (E) Tail tip phenotype scored on 1-day old adults. (F) pharyngeal pumping rate; (G) brood size; (H) life span; (I) defecation rate; (J) locomotion rate. Most paqr-2 mutant phenotypes were suppressed by the paqr-1(et52) gain-of-function mutation, and unaffected or worsened by the paqr-1(tm3262) loss-of-function mutation. The dashed line in D indicates the approximate size of the L1 larvae at the start of the experiment. Significant differences compared to the paqr-2 genotype are indicated where: * p<0.05, **p<0.01 and ***p<0.001 (ns: not significant). (TIFF) [file pgen.1008975.s001.tiff]

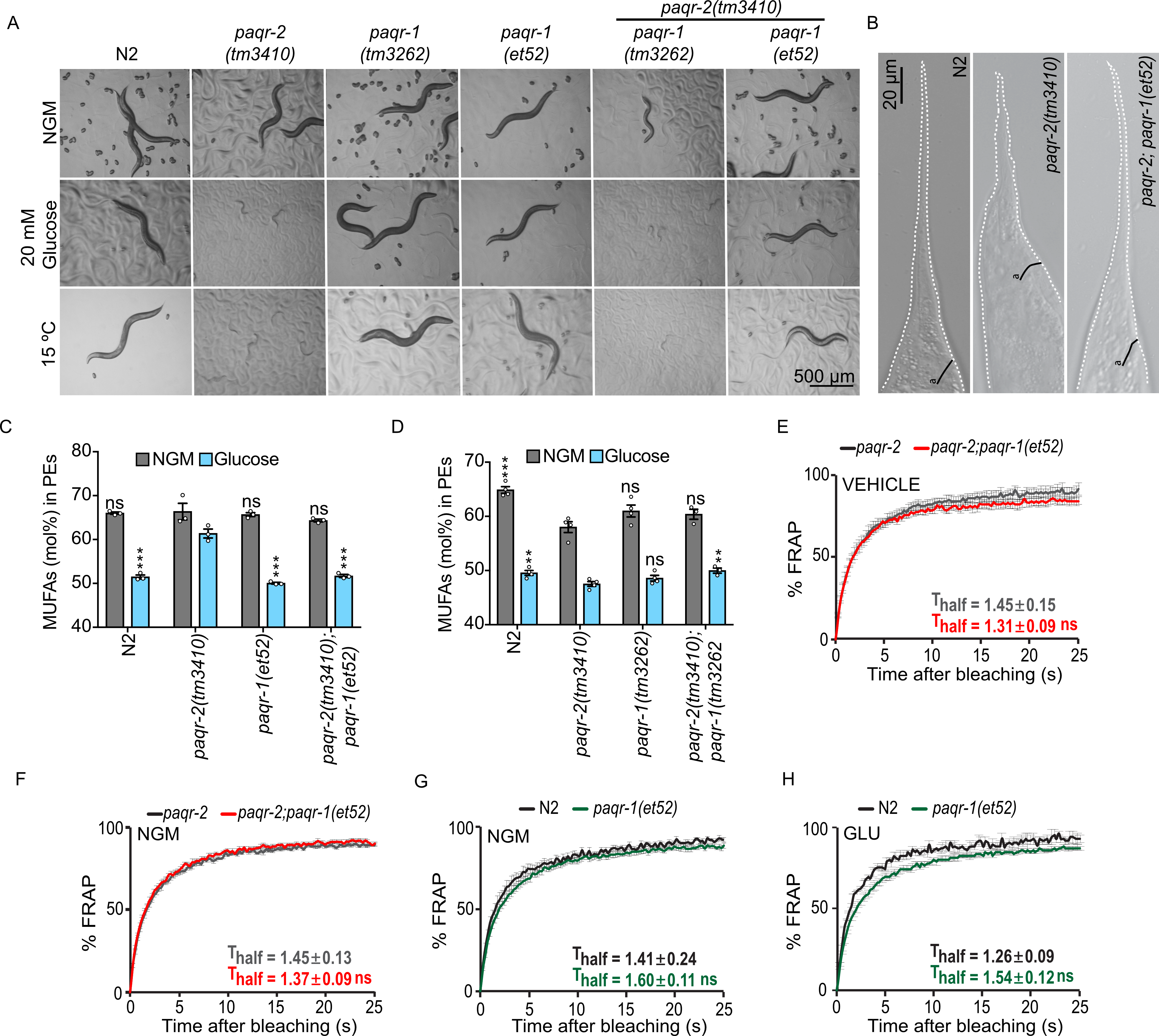

Supplement: S2 Fig — (A) Photographs of worms of the indicated genotypes spotted as L1s then cultivated for 72 hours on NGM plates or on plates containing 20 mM glucose, or 144 hours on NGM plates incubated at 15°C. (B) The paqr-1(et52) alleles suppresses the tail tip defect of the paqr-2 mutant. (C-D) The paqr-1(et52) mutation suppresses the excess MUFA and depletion of MUFAs in the PEs of paqr-2 mutant worms, especially when worms are grown on 20 mM glucose. (E-H) FRAP measurements showing that the gof paqr-1(et52) has no effect on the membrane fluidity of wild-type or paqr-2 mutant worms grown on normal media. Significant differences compared to the paqr-2 genotype are indicated where: * p<0.05, **p<0.01 and ***p<0.001 (ns: not significant). (TIFF) [file pgen.1008975.s002.tiff]

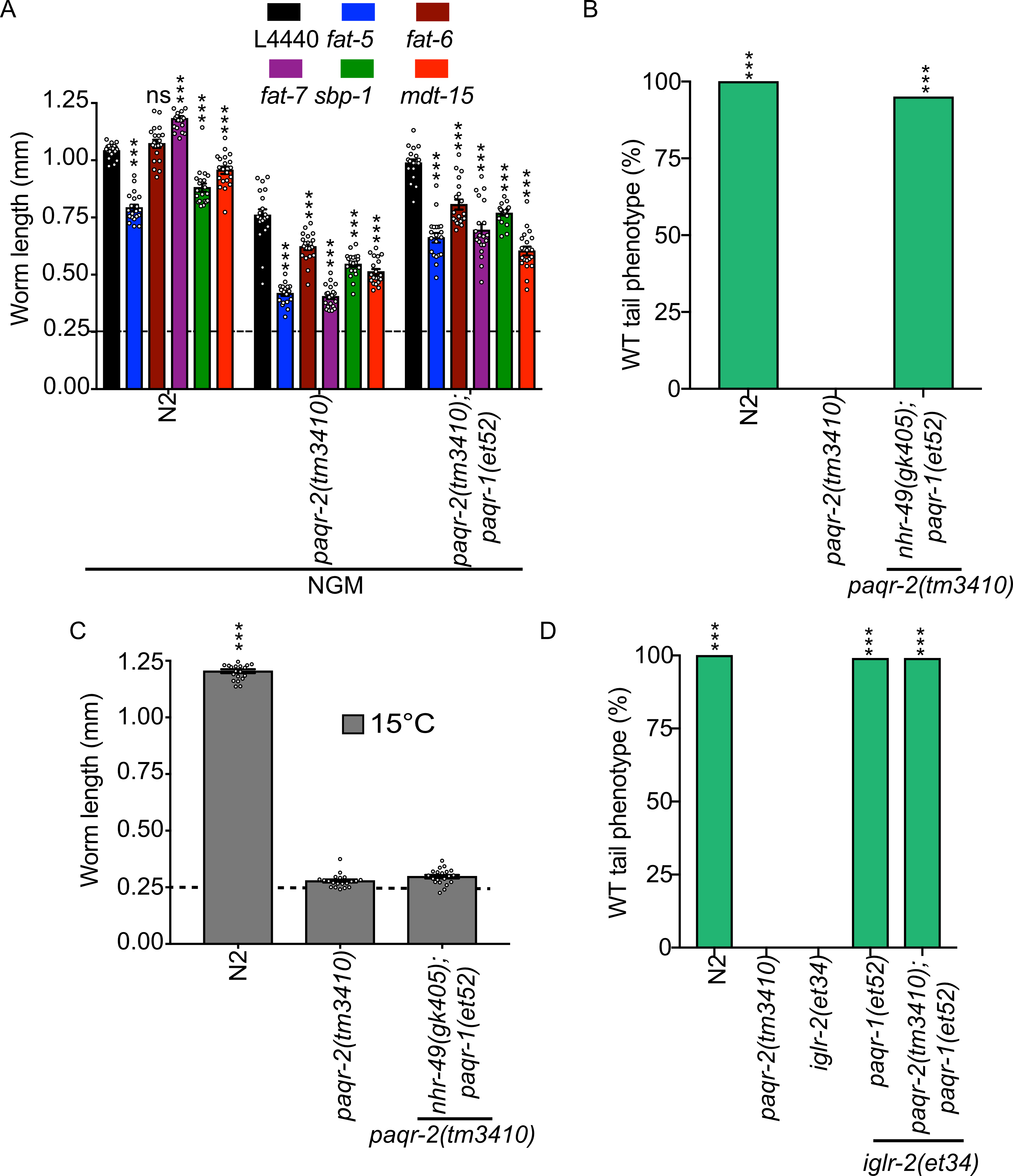

Supplement: S3 Fig — (A) Length of worms placed as L1s on the indicated RNAi treatments and measured 72 hours later. (B) tail tip phenotype scored on 1-day old adults of the indicated genotypes. (C) Length of worms with the indicated genotypes placed as L1s on NGM media and measured after 144 hours cultivation at 15°C. Note that paqr-1(et52) is unable to suppress the cold intolerance of the paqr-2 mutant when nhr-49 is also mutated. (D) Tail tip phenotype scored on 1-day old adults of the indicated genotypes. Note that paqr-1(et52) is able to suppress the cold tail tip defect of the paqr-2 mutant even when nhr-49 or iglr-2 are also mutated. The dashed lines in A and C indicate the approximate size of the L1 larvae at the start of the experiment. Significant differences compared to the paqr-2 genotype are indicated where: * p<0.05, **p<0.01 and ***p<0.001 (ns: not significant). (TIFF) [file pgen.1008975.s003.tiff]

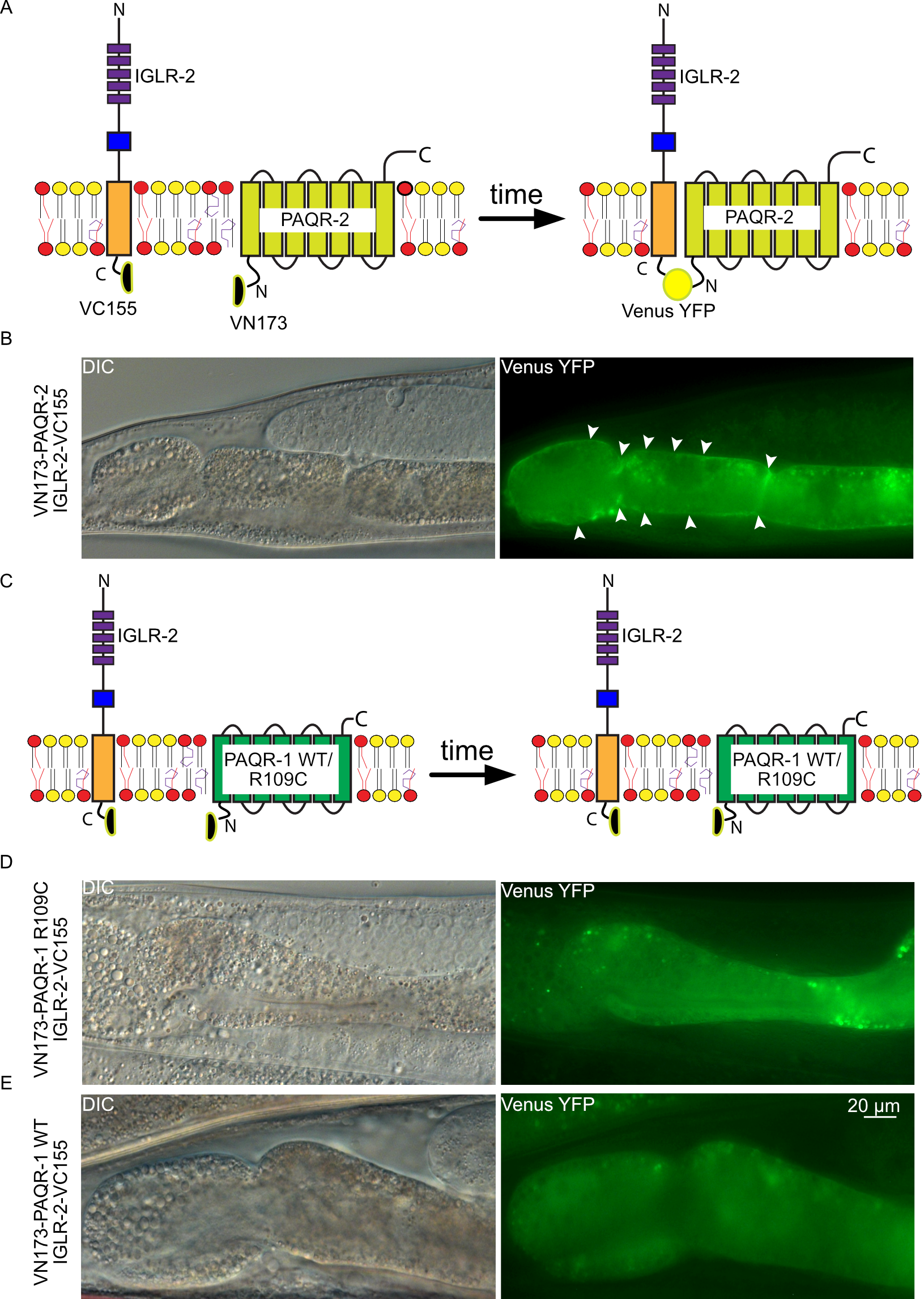

Supplement: S4 Fig — (A) Schematic structures of IGLR-2 fused to VC155 and PAQR-2 fused to the VN173 fragment. BiFC could occur over time if the interaction between the two proteins allows reconstitution of a full and fluorescent VENUS YFP. (B) Visualization of the BiFC signal on the plasma membrane of intestinal cells (arrowheads). (C) Schematic structures of IGLR-2 fused to VC155 and PAQR-1 (wild type or R109C variant) fused to the VN173 fragment. BiFC would not occurs over time if no interaction occurs between the two proteins. (D and E) No BiFC signal was detected on the plasma membrane when using either wild-type PAQR-1 or PAQR-1(R109C) as a possible partner for IGLR-2; the diffuse background signal is due to autofluorescence in the intestine. (TIFF) [file pgen.1008975.s004.tiff]

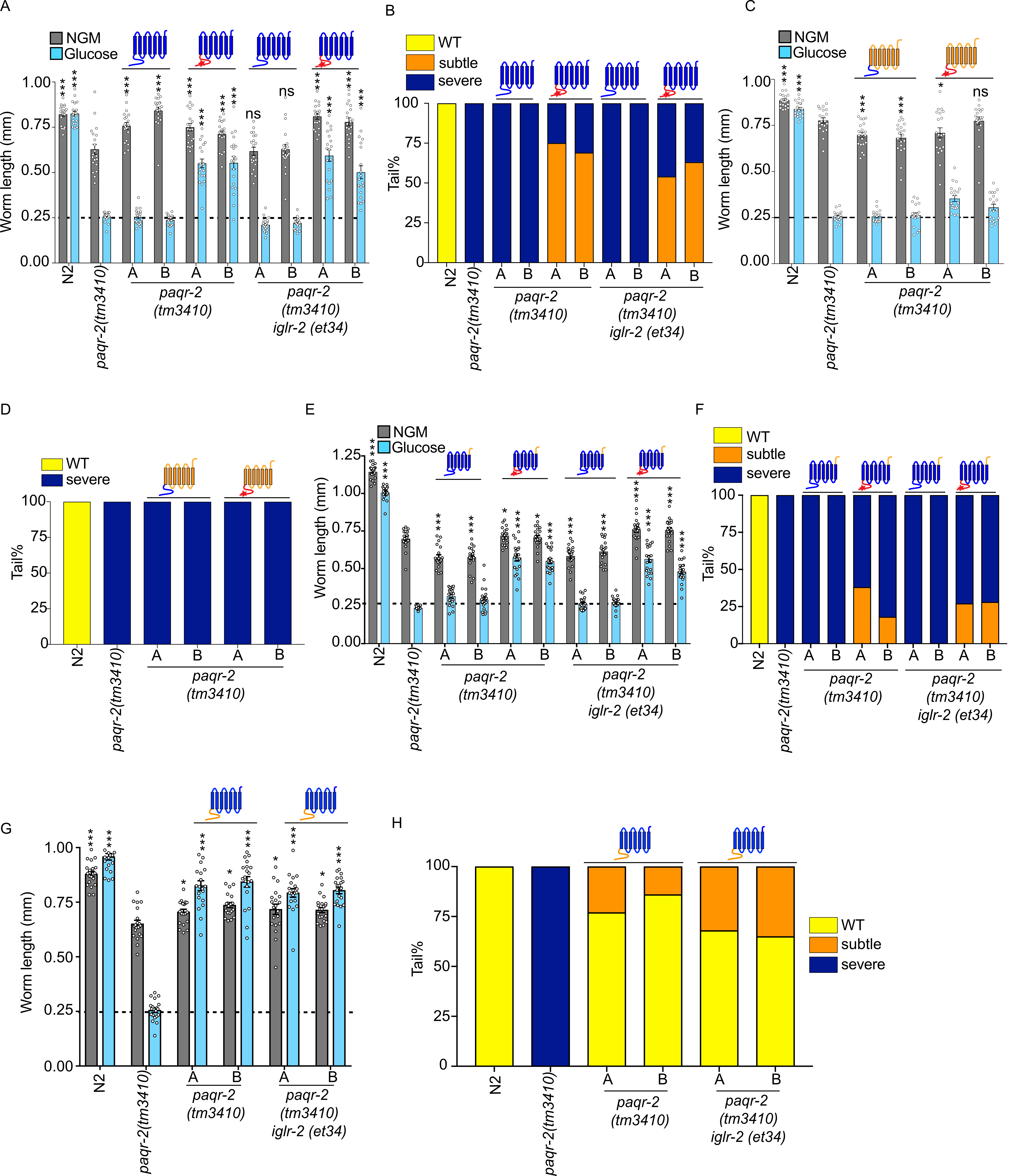

Supplement: S5 Fig — The cartoon representation of the various constructs used in this structure-function study are as in Fig 4A, and A/B indicate two separate transgenic lines for each construct in the indicated genetic background. (A, C, E and G) Length of worms placed as L1s on NGM media and measured after 72 hours of cultivation on NGM plates or plates containing 20 mM glucose. (B, D, F and H) Tail tip phenotype scored on 1-day old adults. The dashed lines in A, C and E indicate the approximate size of the L1 larvae at the start of the experiment. Significant differences compared to the paqr-2 genotype are indicated where: * p<0.05, **p<0.01 and ***p<0.001 (ns: not significant). (TIFF) [file pgen.1008975.s005.tiff]
