# Supplementary material for: Leveraging a gain-of-function allele of Caenorhabditis elegans paqr-1 to elucidate membrane homeostasis by PAQR proteins
Source: PLoS Genet. 2020 Aug 4;16(8):e1008975. doi: 10.1371/journal.pgen.1008975 (PMC7428288; doi:10.1371/journal.pgen.1008975)
Supplement: Attachment — Submitted filename: 200604_Answers_to_Comments.pdf [file pgen.1008975.s007.pdf]

Response to reviewers' comments, PGENETICS-D-20-00388

We thank all three reviewers for their judicious and helpful comments and suggestions. Addressing all of the comments has made this a much better manuscript. Below is a point-by-point answer to the reviewer comments, with changes to the manuscripts clearly indicated in each instance.

Reviewer #1:
This reviewer was very positive regarding our manuscript and found this paper to be well written, the experiments rigorous and well executed, and the findings to be of importance to the field. The conservation with mammalian proteins, together with some experimental data using HEK293 cells demonstrates the importance of these findings to human physiology and medicine.

The reviewer's concerns mainly dealt with writing and presentation. We thank the reviewer for these comments which helped us to greatly improve the manuscript, as follows.

1. In the Introduction, the authors write (line 94), "...we performed a forward genetic screen for enhancers of the ability of mdt-15(et14) to suppress the SFA intolerance of the paqr-2(tm3410) null mutant." However, after that, these mutants are always referred to as suppressors, including in the materials and methods. I understand that the mutants are suppressors of the paqr-2 phenotypes, as is mdt-15(et14), but referring to them as enhancers is confusing here. Please clarify the writing.

We have now clarified the text. We now write (lines 99-101): "Recently, and to identify additional components of the *paqr-2* pathway, we performed a forward genetic screen to isolate mutations that enhance the ability of *mdt-15(et14)* to suppress the SFA intolerance of the *paqr-2(tm3410)* null mutant. Of 15 novel mutants isolated…"

2. On line 162, this latter part of this sentence was confusing. "In contrast, the loss-of-function allele paqr-1(tm3262) has no paqr-2 suppressor effect or indeed significantly worsens paqr-2 mutant phenotypes such as poor growth with/without glucose."

We have now clarified the text as per the reviewer's suggestion (lines 172-175): "In contrast, the loss-of-function allele *paqr-1(tm3262)* does not act as a *paqr-2* suppressor but rather slightly worsens several *paqr-2* mutant phenotypes such as poor growth on normal media (**Fig 2A**), phospholipid composition defects (**Fig 2F-G; S3D Fig**), brood size (**S2F**) and locomotion rate (**S2J Fig**). These results suggest that the wild-type *paqr-1* and *paqr-2* genes are partially redundant for these traits."

3. In the Abstract, end of Introduction, Results, and Discussion, the authors refer to the "downstream effectors", with sentences like: "PAQR-1 does not require IGLR-2 but likely competes with PAQR-2 for downstream effectors". This made me anticipate that downstream proteins interacting with the PAQR-2 complex were identified (perhaps a substrate?). However, as I read the paper I realized that by "downstream effectors" they meant the first steps of the fatty acid desaturation pathway (fat-5,6,7), which are regulated by sbp-1, nhr-49, and mdt-1. That these genes are required for increased fatty acid desaturation downstream of

paqr-2 has been shown previously. This was disappointing, because using the term "downstream effectors" set the reader up for expecting a new discovery.

We have now rephrased every instance where the term "downstream effectors" was previously used, and indicate instead that PAQR-1 and PAQR-2 act via the same pathway.

4. Furthermore, the experiments to show that they are required for paqr-1 (GOF) suppression in Figure 3 were quite weak. RNAi was used, and the exact clones used were not described (Ahringer library?). The authors admitted that the bacterial host for RNAi, HT115, is quite a bad diet for paqr-2 and paqr-2/paqr-1(gof) mutants. In fact, the paqr-1(gof) only very slightly suppresses the paqr-2 under these conditions. Thus, the evidence is a minor change in this severe phenotype, making it slightly worse when the delta-9 desaturases and their regulators are depleted by RNAi. To be more convincing, the researchers need to find a way to do this outside of the HT115 background (some people have developed RNAi in OP50 background, or else they could make one or two triple mutants). RNAi of fat-5, fat-6, and fat-7 is tricky anyway, the Ahringer RNAi clones almost certainly knock down the other paralogs, especially fat-6 and fat-7, which have a very high degree of sequence similarity. In summary, the evidence that paqr-1 and paqr-2 "compete" for "downstream effectors" is very weak.

We agree that RNAi is tricky and that repeating these experiments using OP50 would be an improvement. Aware of such limitations, we did not overinterpret the results obtained, merely pointing out that they do suggest that PAQR-2 and PAQR-1 likely depend on similar downstream genes. Importantly, we also did confirm epistatic interactions with *nhr-49* mutants (Fig. 3C-D). We have now added a M&M section concerning the RNAi experiments (we did use the Ahringer library), and also emphasized that RNAi against the desaturases may be non specific (lines 182-184): "Using RNAi, we found that *mdt-15*, *sbp-1* and the *fat-5/-6/-7* desaturases (which share high sequence similarity and may all be silenced by siRNA against any one)…".

5. In Figure 6, the model is nice and it implies that the PAQR-1 and PAQR-2 are competing for the substrate of the hydrolase. Any speculation as to the substrate? This model emphasizes that the two proteins are not competing for effectors such as fatty acid desaturases or regulatory transcription factors, but for a substrate that might be involved in signaling, confirming the issues with evidence for "competing for downstream effectors" brought up in comment #3.

We now remind the reader that likely substrates for PAQR-2 are ceramides (lines 354-355): "… that result in its activation, i.e. displacement of the "ball" that regulates access of substrates (e.g. ceramides as per [22, 28, 29]) to the active site (see model in **Fig 6**)."

We also clarify our hypothesis concerning how PAQR-1 and PAQR-2 may compete for interactors (legend to Fig. 6): "The model attempts to explain the regulation of the hydrolase activity (e.g. ceramidase) that likely provides a ligand for downstream targets; PAQR-1 and PAQR-2 may have additional common interaction partners that explain why PAQR-2 can act as an inhibitor of PAQR-1(R109C) when IGLR-2 is absent (not shown)."

Reviewer #2
This reviewer was also very positive regarding our manuscript and found it to be "<u> a very interesting and solid manuscript, well written and illustrated, that present important advances</u>

in the mechanism of PAQR type proteins to achieve regulation of membrane homeostasis."

The reviewer also had minor questions and suggestions which we were very thankful for and addressed as follows.

1. One of the conclusions obtained with domain swapping is that the TM domains of PQR-2 dictate the requirement of IGLR-2 for activity, while genetic analysis shows that PAQR-1 is independent of IGLR-2. This is quite surprising since the TM domains of PAQR-1 and PAQR-2 are highly homologous! Do they test if a chimera of the TM domains of PAQR-1 and the entire N-terminal domain of PAQR-2 (rather that the N terminus of PAQR-1 (R109C)) produces an protein that requiring IGLR-2?

We have now generated the suggested chimera (construct 7 in Fig. 4A-B) and added several new figure panels (Fig 4F, S5G-H9). The results with this new construct were great! We now write (lines 252-257): "Finally, a construct bearing the transmembrane domains of PAQR-1 fused to the cytoplasmic N-terminal domain of PAQR-2 was created and found to act as a potent *paqr-2* suppressor both in the presence or absence of IGLR-2 (**Fig. 4F** and **S5G-H Fig**). This is an interesting result from which at least two conclusions may be drawn: 1) it is the transmembrane domains of PAQR-2 that impose a requirement for IGLR-2; and 2) fusing the PAQR-1 transmembrane domains to the cytoplasmic domain of PAQR-2 results in a protein that behaves as a PAQR-1 gain-of-function allele."

And later, in the discussion (lines 332-334): "Such a mechanism is also consistent with the gain-of-function allele created by fusing the transmembrane domains of PAQR-1 to the cytoplasmic domain of PAQR-2, presumed here to poorly inhibit access to the catalytic site."

2. Do the authors have any data of bi-fluorescence assay between IGLR-2 and PAQR-1?
We have now performed new BiFC experiments confirming that IGLR-2 interacts with PAQR-2 on the plasma membrane, but not with either wilt-type PAQR-1 or PAQR-1(R109C). These results are entirely consistent with the expectations from the genetic interactions studies (i.e. PAQR-1(R109C) acts even in a *iglr-2* null mutant background; Fig 4C). This is presented in the new S4 Figure, and in the main text (lines 207-210) where we now write: "Indeed, BiFC, a method that detects the interaction between PAQR-2 and IGLR-2 [17] (**S4A-B Fig**), failed to detect any interaction between IGLR-2 and either wild-type PAQR-1 or PAQR-1(R109C) (**S4C-D Fig**). This again is consistent with the PAQR-1 proteins acting independently of IGLR-2."

3. What is the evidence that PAQR-1 acts constitutively? (Rows 294-295). The genetic data show that this protein is inactive.
We modified the text in the discussion (lines 317-319): "PAQR-1, which does not require IGLR-2, may have a low level of constitutive activity; this would explain the observation that over-expression of the wild-type PAQR-1 partially suppresses *paqr-2* mutant phenotypes."

4. Is there evidence that AdipoR1 and AdipoR2 interacts with a putative IGLR2?
We have so far no evidence either way.

5. In materials and methods are described lipidomic assays that are not mentioned in the text.
The M&M lipidomics section refers to Fig. 2D-G, S2C-D Fig and the S1 Table.

6. Line 286 Discussion. What does mean "what portion was included seem…"?

We now write (lines 305-307): "…the published crystal structures of the AdipoRs does not include the full-length N-terminal domain but the small portion (i.e. residues 89-120 for AdipoR1) that was included seem to obstruct the access to the cytoplasm-facing cavity…"

Reviewer #3:
This reviewer raised several points related to placing the new experiments in the context of earlier findings and suggestions for additional experiments. We thank the reviewer for the comments and have made several improvements to the manuscript.

1. The manuscript by Busayavalasa et al reported the identification of a new gain-of-function allele of paqr-1. Worms bearing the paqr-1(gf) allele suppressed the phenotypes caused by loss of paqr-2 function. These phenotypes include cold intolerance, a reduction in body length, tail morphology defects and intolerance to 20mM glucose. The last phenotype was previously shown by the authors to induce saturated fatty acid accumulation in E. coli, which when consumed by worms, supposedly increased membrane rigidity that was incompatible with the loss of paqr-2 function. In contrast, how did the loss of paqr-2 function reduce the body and affect tail morphology remained mysterious, despite a series of papers from the same lab. The lack of clarity in the casual relationship between paqr-2(lf) and the phenotypes scored made the interpretation of the effect of the new paqr-1(gf) allele difficult.
We now write (lines 86-88): "These phenotypes are secondary to the primary membrane fluidity defects of *paqr-2* and *iglr-2* mutants because they can be suppressed fully or partially by low, fluidizing concentrations of mild detergents [18], by providing supplements of unsaturated fatty acids [16, 18], or by secondary mutations that increase the relative abundance of unsaturated fatty acids (UFAs) among phospholipids, such as *mdt-15(et14), nhr-49(et8), fld-1(et48)* and several others [14, 17, 18]. PAQR-2 and IGLR-2 mostly act cell non-autonomously: expression in one large tissue (e.g. gonad sheath cells, intestine or hypodermis) is sufficient to rescue the entire worm; the one exception is the tail tip which requires local expression likely because it constitutes a site of poor lipid exchange with the rest of the worm [15]."

2. The expression pattern of PAQR-1 was described mostly in words instead of being shown (Figure 1D).
We now provide better images of the expression in gonad sheath cells and intestine (Fig. 1D). We write (lines 133-136): "The localization of GFP translational reporters is also unchanged between the wild-type PAQR-1 and the mutant PAQR-1(R109C) proteins, which are both expressed in several tissues, but predominantly in the intestine and gonad sheath cells, which is the site of strongest PAQR-2 expression [17] **(Fig 1D)**."

3. No attempt was made to demonstrate the site of action of PAQR-1.
This is true but, as just mentioned, we do show that PAQR-1 is expressed predominantly in the intestine and gonad sheath cell (also the predominant site of PAQR-2 expression). See also answer to the first comment from this reviewer regarding the cell non-autonomous actions of PAQR-2, which was the focus of an earlier publication (ref 15 in the manuscript).

4. Although the authors claimed that "paqr-1(et52) suppresses all paqr-2 mutant phenotypes" (Line 149), the results on body length when worms were grown on glucose containing plates suggested otherwise (Figure 3B).
We now write (Lines 158-162): "***paqr-1(et52)* suppresses *paqr-2* mutant phenotypes.** Having established, using transgenic animals, that *paqr-1(et52)* is a *gof* allele, we proceeded to its more detailed characterization. The *paqr-1(et52)* mutation suppresses, partially or

entirely, the defects in pharyngeal pumping rate, brood size, lifespan, defecation rate, and locomotion rate of the *paqr-2* mutant (**S2F-J Fig**). Indeed, the *paqr-1(et52)* mutation at partially or entirely suppresses all *paqr-2* defects tested so far, including…" Incidentally, the combination of the HT115 dietary bacteria used in RNAi experiment together with glucose, as used in Fig 3B, is potently toxic for the *paqr-2* mutant and explains the weaker effect of *paqr-1(et52)* in that condition.

5. The claim that "the intolerance to SFA, which is a harder challenge for the paqr-2 mutant" (Line 135) was also hard to understand.
We now clarify this statement as follows (lines 142-144): "intolerance to SFA, which is a harder challenge for the *paqr-2* mutant since many *paqr-2* suppressor alleles that fully rescue growth at 15°C do not effectively rescue growth on glucose [14, 18, 35](**Fig 1E; S1A-C Fig**)."

6. In the absence of a concrete molecular explanation, it is impossible to interpret the way different chimeric PAQR-1/PAQR-2 proteins functioned, in transgenic worms that were studied in various assays (Figure 4 and Figure S4). Therefore, the assignment of specific functions to the N-terminal cytosolic domain and the transmembrane domain of PAQR-2 and PAQR-1 was premature.
As mentioned above (Reviewer 2, point 1), we have now generated the suggested chimera (construct 7 in Fig. 4A-B) and added several new figure panels (Fig 4F, S5G-H), and now write (lines 252-257): "Finally, a construct bearing the transmembrane domains of PAQR-1 fused to the cytoplasmic N-terminal domain of PAQR-2 was created and found to act as a potent *paqr-2* suppressor both in the presence or absence of IGLR-2 (**Fig. 4F** and **S5G-H Fig**). This is an interesting result from which at least two conclusions may be drawn: 1) it is the transmembrane domains of PAQR-2 that impose a requirement for IGLR-2; and 2) fusing the PAQR-1 transmembrane domains to the cytoplasmic domain of PAQR-2 results in a protein that behaves as a PAQR-1 gain-of-function allele." The results with this new chimera are consistent with our earlier interpretations. We think that we can conclusively state that the transmembrane domains of PAQR-2 are responsible for its requirements for IGLR-2. We agree that assigning a regulatory function to the cytoplasmic domains of PAQR-1 and PAQR-2 remains speculative, though consistent with our result, which we point out in the manuscript.

7. The genetic interaction studies on paqr-2(lf), iglr-2(lf) and paqr-1(gf) was insufficient to formulate the model proposed in Figure 6. The authors have previously used BiFC assays to demonstrate the interaction between PAQR-2 and IGLR-2. Similar molecular interaction studies are essential to place PAQR-1 in the model.
The model in Fig. 6 is admittedly speculative (as we write in the text) but consistent with our new structure-function studies. Its speculative nature serves the purpose of providing a clear framework for future experiments. Additionally, it is clear that PAQR-1 does not interact with IGLR-2 based on several observations. They do not interact physically as previously shown using BiFC (PLoS Genetics 2016), and repeated here now also for the gain-of-function PAQR-1(R109C) (see the new BiFC data presented in the new S4 Fig). Also, we know that wild-type PAQR-1 is still active in the absence of the IGLR-2 protein since the *iglr-2* mutant is less severe that the *paqr-1;paqr-2* double mutant (PLoS One 2011 and the present work). Finally, neither the PAQR-1(+) nor PAQR-1(gof) require IGLR-2 for their activity when provided as transgenes, as we have shown and emphasized repeatedly in the present work.

8. The speculation on the role of the R109C gain of function mutation in PAQR-1 was also tenuous. The authors ruled out the possibility of aberrant disulfide bridges involving the extra cysteine residue. Instead, they proposed that the arginine residue was crucial to hold the N-terminal hypothetical regulatory domain in position, according to the model in Figure 6. If that is the case, the deletion of the entire N-terminal domain of PAQR-1 should provide the strongest suppression of paqr-2(lf) mutant phenotypes.
This is an excellent suggestion from the reviewer. As mentioned above (reviewer 2, point 1), the new data on fusing the cytoplasmic domain of PAQR-2 to the transmembrane domains of PAQR-1 produced a gain-of-function allele that acts independently of IGLR-2. This add important support for the notion that the cytoplasmic domain is regulatory since swapping it changes the activation state of the chimeric protein. Regarding future experiments, we now write (lines 312-315): "It will be interesting in the future to test whether novel gain-of-function alleles may be created by expressing PAQR-1 or PAQR-2 with truncations in their cytoplasmic N-terminal domains, which incidentally harbors membrane localization motifs in the mammalian AdipoRs [39, 40]."

9. The results on the effects of overexpressing mammalian AdipoR1 or AdipoR2 in HEK293 cells were impossible to interpret. This is because the authors did not demonstrate if PAQR-1 and PAQR-2 are functional homologs of AdipoR1 and AdipoR2, respectively.
We actually did show in a recent publication that AdipoR1 and AdipoR2 are functional homologs of PAQR-2. We now write (lines 266-269): "For this purpose, we turned to HEK293 cells where the AdipoR1 and AdipoR2 proteins act as functional homologs of PAQR-2 and are required to prevent membrane rigidification when the cells are cultivated in the presence of palmitic acid [13-16, 35]."

10. Nor did they show if the expression of PAQR-1 in mammalian cells would trigger the same effect on membrane fluidity.
This is an interesting suggestion, though a concern is that the mammalian and *C. elegans* proteins may be optimized for quite different temperatures, which is of special relevance for membrane fluidity regulators important during temperature adaptation.

11. The similarity in primary amino acid sequence is not a guarantee of functional similarity.
We completely agree but as just mentioned we have shown that they are functionally similar.
